# Epidemiological survey and genetic characterization of type 3 vaccine-derived poliovirus isolated from a patient with four doses of inactivated polio vaccine in Henan Province, China

**DOI:** 10.1186/s40249-022-01028-1

**Published:** 2022-12-14

**Authors:** Mingyu Zhang, Jianhui Yang, Yiran Bai, Hui Zhu, Changshuang Wang, Lu Zhang, Jin Xu, Mingxia Lu, Xiaoxiao Zhang, Zhanpei Xiao, Yating Ma, Yan Wang, Xiaolei Li, Dongyan Wang, Shuangli Zhu, Dongmei Yan, Wenbo Xu, Yong Zhang, Yanyang Zhang

**Affiliations:** 1 Henan Center for Disease Control and Prevention, Zhengzhou, Henan People’s Republic of China; 2 WHO WPRO Regional Polio Reference Laboratory and National Health Commission Key Laboratory for Biosafety, National Institute for Viral Disease Control and Prevention, Chinese Center for Disease Control and Prevention, Beijing, People’s Republic of China; 3 Center for Biosafety Mega-Science, Chinese Academy of Sciences, Wuhan, 430071 People’s Republic of China

**Keywords:** Epidemiological Survey, Genetic characterization, Type 3 vaccine-derived poliovirus, Recombination, Henan, China

## Abstract

**Background:**

Vaccine-derived poliovirus (VDPV) is a potential threat to polio eradication because they can reintroduce into the general population and cause paralytic polio outbreaks, a phenomenon that has recently emerged as a prominent public health concern at the end of global polio eradication. This study aimed to describe the epidemiology and genetic characteristics of the first VDPV identified from a patient with acute flaccid paralysis (AFP), with four doses of inactivated polio vaccine immunization in Henan Province, China in 2017.

**Methods:**

The patient was diagnosed with type 3 VDPV. Subsequently, a series of epidemiological approaches was implemented, including a retrospective search of AFP cases, rate of vaccination assessment, study of contacts, and supplementary immunization activities. Fecal samples were collected, viral isolation was performed, and the viral isolates were characterized using full-length genomic sequencing and bioinformatic analysis.

**Results:**

Phylogenetic analysis showed that the viral isolates from the patient were different from other reported genetic clusters of type 3 VDPV worldwide. They were identified as a Sabin 3/Sabin 1 recombinant VDPV with a crossover site in the *P2* region. Nucleotide substitutions, including U → C (472) and C → U (2493), have been identified, both of which are frequently observed as reversion mutations in neurovirulent type 3 poliovirus. A unique aspect of this case is that the patient had been vaccinated with four doses of inactive polio vaccine, and the serum neutralizing antibody for Sabin types 1 and 3 were 1∶16 and 1∶512, respectively. Thus, the patient was speculated to have been infected with type 3 VDPV, and the virus continued to replicate and be excreted for at least 41 d.

**Conclusions:**

The existence of this kind of virus in human population is a serious risk and poses a severe challenge in maintaining a polio-free status in China. To the best of our knowledge, this is the first report of VDPV identified in the Henan province of China. Our results highlight the importance of maintaining a high-level vaccination rate and highly sensitive AFP case surveillance system in intercepting VDPV transmission.

**Supplementary Information:**

The online version contains supplementary material available at 10.1186/s40249-022-01028-1.

## Background

Owing to the success of worldwide vaccination campaigns, the burden of poliomyelitis has radically decreased [1]. In October 2000, China and other countries in the World Health Organization (WHO) Western Pacific Region were polio-free. However, the risk of importation of wild-type poliovirus (WPV) still exists in these countries, such as type 1 WPV importation to the mainland of China in 2011[2]. Another risk is the emergence of vaccine-derived poliovirus (VDPV) that mutated from live-attenuated oral polio vaccine (OPV) strains [2–4].

The genetic variability of polioviruses is mostly due to nucleotide substitutions and recombination [5–7]. Owing to the inherent genetic instability of polioviruses, OPV (a live attenuated poliovirus) also undergoes frequent mutations throughout its genome during replication in the human intestine. OPV has many advantages in polio eradication, including ease of administration, efficient induction of intestinal immunity, triggering of durable humoral immunity, and low cost. However, wider application of OPV is limited by genetic instability, a potential cause of vaccine-associated paralytic poliomyelitis (VAPP), and the emergence of genetically divergent VDPVs [8, 9]. VDPVs are defined as OPV-related isolates that differ from those of the parental strain by 1%–15% for serotypes 1 and 3, and 0.5%–15% for serotype 2 in the viral protein 1 (*VP1)* coding region. VDPVs have the potential for sustained circulation in areas with low OPV immunization, and many outbreaks of circulating VDPVs (cVDPVs) have been reported worldwide in recent years [10–12]. After vaccination with OPV or infection with OPV-related virus in immunodeficient patients, especially patients with B-lymphocyte immunodeficiency, immunodeficient VDPV (iVDPV) is produced due to the long-term existence of vaccine in the body, continuous replication of the virus, and viral excretion from the body [13, 14]. If neither cVDPV nor iVDPV is evident, such VDPV is classified as ambiguous VDPV (aVDPV) [15].

In this study, we reported an acute flaccid paralysis (AFP) case with four doses of inactive polio vaccine in Runan County, Zhumadian City, Henan Province, in February 2017. Although the patient was diagnosed with viral myositis by a provincial Polio Expert Committee after a 60-day follow-up physical examination, this case was diagnosed as VDPV patient due to the isolated type 3 VDPV, in accordance with the latest recommendations from the WHO [16]. This report described the results of epidemiological and laboratory investigations and the actions taken to prevent the circulation of VDPV.

## Materials and methods

### Case investigation

Cases of paralytic polio were identified by an AFP case surveillance system in China. A AFP patient was reported in Henan Province in 2017, the patient was born on 12 September 2014, developed symptoms of right leg weakness and right lower limb claudication without obvious inducement on 14 February 2017, and continued to worsen and had difficulty walking on 16 February, and was admitted to Zhumadian Central Hospital as an AFP case and reported.

A series of fecal samples were collected from a patient on February 23, February 24, April 5, April 23, May 3, May 10, and May 19, 2017. After the fecal samples were sent to Henan Provincial Polio Laboratory, according to the polio laboratory manual, human rhabdomyoma cell (RD cell) and mouse cell line expressing the gene for human cellular receptor for poliovirus (L20B cell) were used for viral isolation [17], and intratypic identification (ITD) and VDPV screening were performed using real-time polymerase chain reaction (rRT-PCR) method [18], of which L20B cell-positive isolates were isolated from the first three fecal samples. After L20B cell positive isolates were submitted to the National Polio Laboratory, they were identified as type 3 VDPV using *VP1* region nucleotide sequencing method [4]. To investigate the scope of the virus epidemic, after VDPV was identified, investigations among 19 relatives and 20 close contacts who had contact with the patient were performed by testing stool samples for poliovirus. The vaccination certificates of the individuals who had contact with the patient were examined.

AFP cases were actively searched and crosschecked using the records of all county- and prefecture-level hospitals in Zhumadian City, Henan province. To prevent possible circulating VDPV, supplemental immunization activities (SIAs) were conducted throughout the county. House-to-house SIAs were conducted in urban regions.

Humoral immunity function was measured using blood samples from the patient collected on April 21, 2017. The neutralizing antibodies for immunoglobulin G of serotypes 1 and 3 were determined using a microneutralization assay with authentic Sabin strains, in accordance with the WHO guidelines in the Henan provincial Center for Disease Control and Prevention (CDC). A serum sample was considered positive if the neutralizing antibody level was determined at a dilution of ≥ 1∶4.

### Detection of viruses in stool specimens collected from AFP cases and in surrounding environment

The child lived in Xiaowan Village, Runan County, Zhumadian City, after VDPV was identified, three sewage samples (1 L each) were obtained via grab sampling from local environmental water sources, including household wastewater and nearby river water. The samples were immediately transferred to the laboratory under refrigerated conditions. The 1-L sample was concentrated through improved negative-charge filter membrane absorption, and then the virus was eluted in 10 ml of 3% beef extract solution (pH 9.6) after sonication and centrifugation [19]. Thereafter, 200 μl of each concentrated eluent was used to inoculate L20B and RD cells for virus isolation.

Stool specimens collected from the AFP patient, 19 relatives and 20 close contacts of the AFP patient were forwarded to the Henan Provincial Polio Laboratory, where viral isolates were obtained from L20B and RD cell cultures. L20B cell-positive isolates underwent ITD and VDPV screening using rRT-PCR method (Poliovirus rRT-PCR ITD/VDPV-V5.0 kits, Centre for Disease Control and Prevention, USA) to determine whether the isolates were wild or of vaccine origin [18, 20].

Positive isolates from fecal samples and sewage samples were used for RNA extraction. Total RNA was extracted from 140 ml of the infected cell culture using the QIAamp Viral RNA Mini Kit (Qiagen, Valencia, CA, USA), according to the manufacturer’s recommended procedure. The entire *VP1* region of the poliovirus isolates was amplified using RT-PCR with primers that flanked the *VP1*-coding region (upstream primer UG1: 5′-GGGTTTGTGTCAGCCTGTAATGA-3′, downstream primer UC11: 5′-AAGAGGTCTCTRTTCCACAT-3′), and the amplicons were bidirectionally sequenced using the ABI PRISM 3130 Genetic Analyzer (Applied Biosystems, Foster City, CA, USA). PCR products were sequenced in both directions to avoid possible ambiguous nucleotides.

### Full-length genome sequencing of the VDPV strains

The full-length genome of the Henan isolates was amplified using two long-distance PCR reactions using the TaqPlus Precision PCR system (600,212, Agilent, Santa Clara, California, USA). The primer pairs Y7/7500A and 0010S48/Q8 were used to amplify the 5.5 kb and 3.5 kb fragments, respectively [21]. The combined sequences of the two fragments yielded the full-length genome sequence. The PCR products were purified for sequencing using the QIAquick Gel Extraction Kit (28,706, Qiagen, Duesseldorf, Germany), and the amplicons were sequenced on the ABI 3130 Genetic Analyzer (Applied Biosystems) as described above. The 5′ end of the genome was amplified using the 5′-Full RACE Kit (6107, Takara, Shiga, Japan) according to the manufacturer’s instructions. The 3′ end of the genome was amplified using an oligo-dT primer (7500A), as previously reported [22].

### Bioinformatics analysis based on the full-length genomic sequences of Henan type 3 VDPVs

Full-length genomic sequences of the Henan VDPV strains obtained in this study were aligned with other type 3 VDPV strains using the ClustalW algorithm implemented in Molecular Evolutionary Genetics Analysis (MEGA) program (version 11.0, The Pennsylvania State University, PA, USA) [23]. All sequences of type 3 VDPV on GenBank (as of May 2022) were downloaded for evolutionary analysis, which were Russia cVDPVs circulating in 2014 (GenBank accession numbers: MT645947–MT645951) [12], iVDPV found in Iran in 2006 (GenBank accession numbers: EU684056–EU684057) [24], aVDPV found in Belarus in 2000 (GenBank accession numbers: FJ460226–FJ460227) [25], aVDPV found in India in 2008 (GenBank accession number: KR259358) [26] and aVDPV found in Hubei province of China in 2014 (GenBank accession number: KY703697). Maximum likelihood trees (based on the *P1*-coding region) were constructed using the GTR + I + G model suggested by jModelTest2 [27] and performed in MEGA program (version 11.0) with 1000 bootstrap replicates. The tree was rooted in Sabin 3 sequence (AY184221). Maximum likelihood trees were also constructed using RAxML (version 8.2.10, Heidelberg Institute for Theoretical Studies, Heidelberg, Germany) to verify the best tree topology [28].

The complete genome sequences of Henan type 3 PV strains were compared with Sabin reference strains (Sabin 1: AY184219, Sabin 2: AY184220, and Sabin 3: AY184221) by pairwise alignment using the MEGA program (version 11.0) [23]. Similarity plots and bootscanning analyses were performed using SimPlot software (version 3.5.1) [29]. Boot scanning analysis was performed using the neighbor-joining method by moving 200 nucleotide windows in 20 nucleotide steps. The recombination event was proved by the phylogenetic tree using neighbor-joining method based on regions before the crossover site (nt1-nt4899) and after the crossover site (nt4900-nt74 31).

The amino acid sequences within the predicted neutralizing antigenic (NAg) sites were aligned with those of the Henan type 3 VDPV strains, Sabin 3 strain (GenBank accession No. AY184221), and type 3 wild poliovirus prototype strain P3/Leon/37 (GenBank accession No. K01392). The key neurovirulence determining sites in Henan aVDPV were determined by full-length genome sequencing analysis.

## Results

### VDPV case investigation

The two-year-old boy was born on August 19, 2014. He received four scheduled doses of inactive polio vaccine (IPV) (at 4, 5, 6, and 18 months) and completed routine polio immunization. His last vaccination date was March 14, 2016. On February 14, 2017, the patient experienced weakness and claudication of the right lower extremity. On February 16, his condition deteriorated with fever (37.5 °C–38.5 °C), diarrhea, and difficulty in walking. The patient was sent to a hospital in Zhumadian City on 16 February 2017, according to the patient 's unique claudication of the right lower limb, low muscle strength, normal muscle tone, presence of physiological reflexes, and negative Barthel sign of the right lower limb, the patient was diagnosed with AFP. Nutritional myocardial and comprehensive rehabilitation treatments were administered after the diagnosis of viral myositis upon admission. The patient gradually recovered with regained strength in the right lower extremity and no residual paresis. On March 12, the fully-recovered patient was discharged from the hospital with a discharge diagnosis of viral myositis.

Viruses were isolated from the first two stool samples collected during hospitalization (February 23 and 24, 2017) and were identified as poliovirus serotype 3 Sabin-like using rRT-PCR. However, sequencing of the *VP1* region of the virus revealed nine nucleotide changes consistent with VDPV3 classification. After the patient was discharged from the hospital, type 3 VDPV was detected again in the third stool sample collected during follow-up (April 5, 2017), with 10/900 (1.11%) nucleotide substitutions in the *VP1* region compared with the type 3 Sabin strain. These three viral strains (CHN21006-1, CHN21006-2, and CHN21006-3) isolated at three time points shared eight nucleotide substitution sites. In the stool samples collected from the fourth to the seventh time periods (April 23, May 3, May 10, and May 19, 2017), the viral isolation results were negative, and no virus was detected. Therefore, it is considered that the excretion of poliovirus has been terminated.

The results of humoral immunity function showed that the serum neutralizing antibody for Sabin type 1 was 1∶16 and Sabin 3 was 1∶512 (1:4 is considered positive), while antibodies against hepatitis B, hepatitis C, and HIV were all negative. Based on all the results, the patient was diagnosed with VDPV classified as VDPV.

### Retrospective analysis of AFP cases and assessment of vaccination coverage

Medical records for both inpatients and outpatients were also reviewed in 23 county-level hospitals in Zhumadian. No additional AFP cases were found during active case searches in hospitals, and no additional AFP cases were observed during house-to-house searches in the villages and neighboring areas.

Through a vaccination information management system for children, the inoculation clinic A and adjacent inoculation clinic B were investigated. The vaccination rate of the third dose of polio vaccine for children under 1 year of age was 80.5%, while the rate of the fourth dose was only 68.93% in vaccination clinic A. The vaccination rates of the second and third doses of polio vaccine were 80.24% and 74.47% in vaccination clinic B respectively. The rate of complete polio vaccination in children under 5 years of age was above 97% in vaccination clinic B.

### Phylogenetic relationship between Henan aVDPV isolates and type 1, type 2, and type 3 VDPV strains

**Fig. 1:**
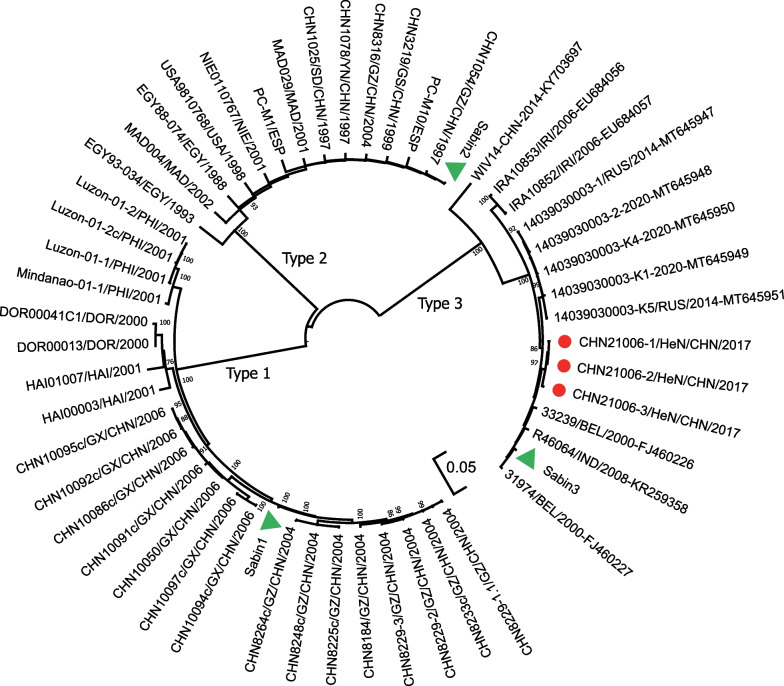
Maximum-likelihood tree showing the phylogenetic relationship between Henan aVDPV isolates and type 1, type 2, and type 3 VDPV strains. The evolutionary history based on the P1-coding region was inferred using the Maximum Likelihood method and Kimura 2-parameter model conducted in MEGA 11. Henan VDPVs are represented by red solid circles and Sabin strains are represented by green solid triangles. *HeN* Henan, *GZ* Guizhou, *GX* Guangxi, *SD* Shandong, *YN* Yunnan, *GS* Gansu; *CHN* China, *RUS* Russia, *USA* The United States. *HAI* Haiti, *DOR* Dominica, *PHI* The Philippines, *EGY* Egypt, *NIE* Nigeria, *ESP* Spain, *MAD* Madagascar, *IRI* Iran, *BEL* Belgium, *IND* India

To elucidate the divergence and evolution of type 3 Henan aVDPV, a maximum-likelihood tree of sequence relationships in the entire *P1* capsid region for Henan type 3 VDPVs and a set of divergent type 1, type 2, and type 3 VDPVs was constructed (Fig. 1).

The phylogenetic tree revealed that all three Henan type 3 VDPVs could be grouped into a single cluster with divergence pathways different from those of Sabin 3, and they were distinct from the genetic clusters of other representative type 3 VDPVs (Fig. 1).

### Recombination features of Henan type 3 VDPVs

**Fig. 2:**
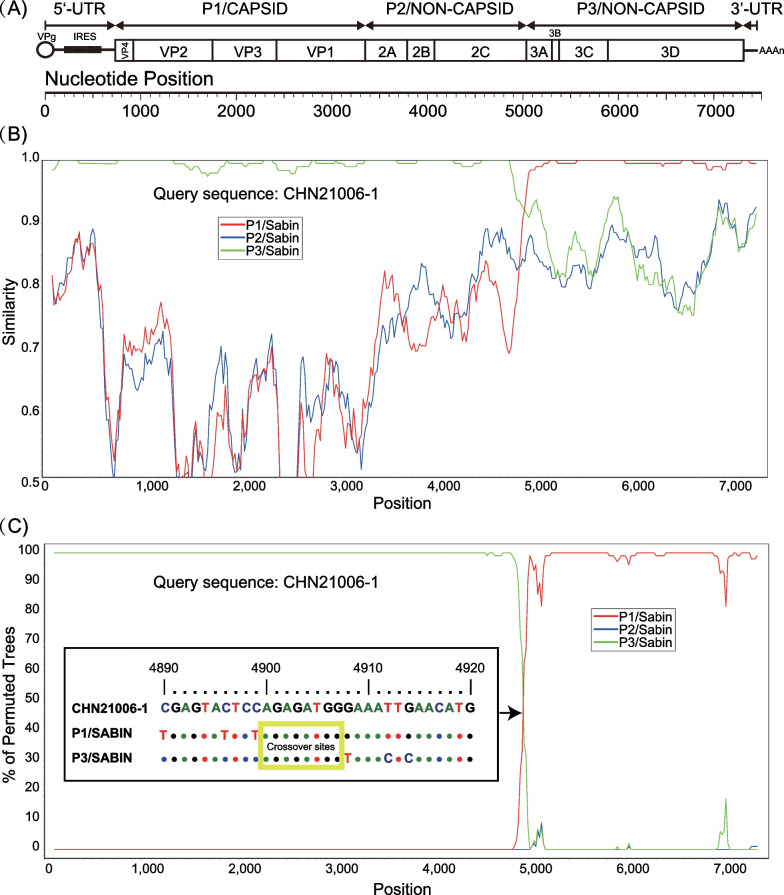
Similarity plot and bootscanning analysis of complete genomes of Henan type 3 VDPVs. Genomic structure organization (**a**), similarity plot (**b**), and bootscanning analysis (**c**) of Henan type 3 VDPV genomes using a sliding window of 200 nt moving in 20 nt steps. Strain CHN21006-1/HeN/CHN/2017 was used as a query sequence and is indicated in the upper left corner. The yellow solid line rectangle indicates possible crossover site

A similarity plot and bootscanning analysis of the complete genomes of the three Henan type 3 VDPVs indicated that they were Sabin 3/Sabin 1 recombinants (Fig. 2). The upstream sequences [i.e., 5′- untranslated region (UTR), P1/capsid, and 5′ part of the P2/noncapsid sequences] up to nucleotide position 4899 were derived from the Sabin 3 strain (28, 30, and 38 nucleotide substitutions, respectively), and sequences downstream of nucleotide position 4899 (i.e., 3′ part of the P2/noncapsid sequences, P3/noncapsid sequences, and 3′-UTR) were derived from the Sabin 1 strain (13, 13, and 22 nucleotide substitutions, respectively) (Fig. 2).

**Fig. 3:**
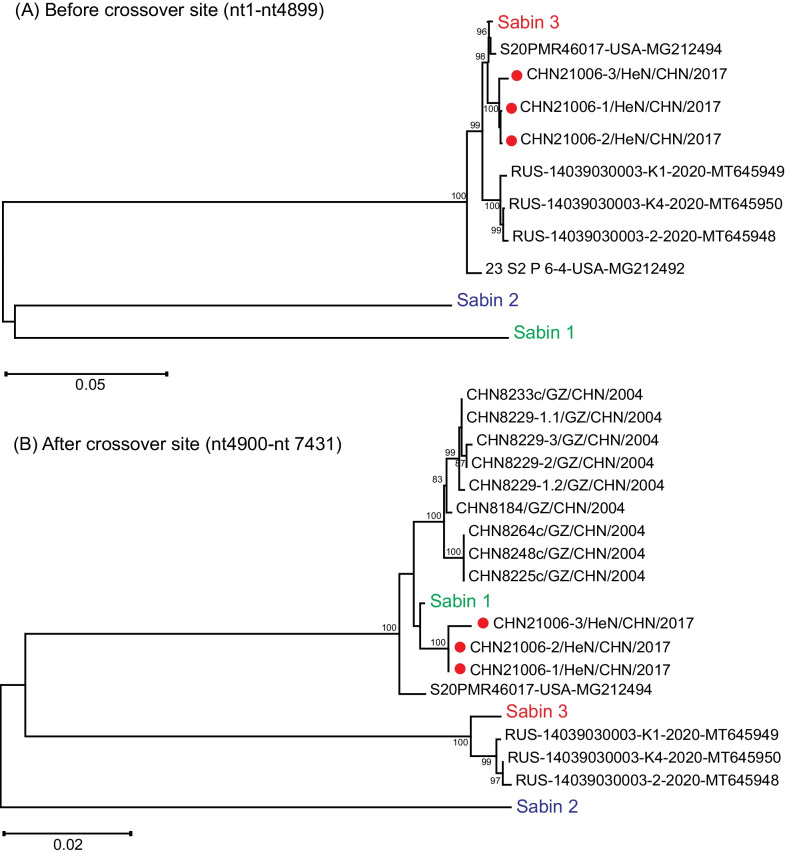
Phylogenetic trees of various genomic sequences constructed based on regions before and after crossover site of Henan type 3 VDPV. **A** Phylogenetic tree constructed based on regions before the crossover site (nt1-nt4899) of Henan aVDPV; **B** Phylogenetic tree constructed based on regions after the crossover site (nt4900-nt7431) of Henan aVDPV. Henan type 3 VDPVs are represented by red solid circles. *HeN* Henan, *GZ* Guizhou, *CHN* China, *RUS* Russia, *USA* The United States

Then the phylogenetic trees of various genomic sequences [three type 3 cVDPVs in Russia (GenBank accession Nos. MT645948–MT645950) and one type 3 aVDPV in USA (GenBank accession No. MG212494)] were constructed based on regions before and after crossover site of Henan type 3 VDPV (Fig. 3). The results showed that Henan VDPV clustered with Sabin 3, three Russian cVDPVs and one American aVDPV in the evolutionary tree constructed based on the sequence of the region before the recombination site, but Henan VDPV clustered with Sabin 1 and one American aVDPV in the evolutionary tree constructed based on the sequence of the region after the recombination site, and three Russian VDPVs still clustered with Sabin 3. This result suggests that the region sequence of Henan VDPV before the crossover site belongs to type 3, while the region sequence after the crossover site belongs to type 1, which confirming the occurrence of recombination. While one American aVDPV sequence had a similar recombination pattern to Henan VDPV, three Russia VDPVs were non-recombinant strains.

### Reversions of key neurovirulence determination sites

Based on the full-length genome sequencing analysis, CHN21006-1, CHN21006-2, and CHN21006-3 contained 41, 43, and 60 nucleotide substitutions, respectively, compared to Sabin3/Sabin1 strain (Yellow labeled nucleotides in Additional file 1: Table S1). Most of the nucleotide substitutions in the coding region of the genome were synonymous, resulting in 9, 9, and 12 amino acid substitutions, respectively (Yellow labeled amino acids in Additional file 1: Table S1).

Notably, the nucleotide U at position 472 in the 5′ noncoding region was mutated to C. Moreover, the nucleotide C at position 2493 in the *VP1* coding region was mutated to U, causing an amino acid substitution in VP1–6: Thr to Ile. Both mutations are neurovirulent reversion mutations usually observed in VDPV-associated paralytic poliomyelitis cases [30]. However, the third nucleotide substitution identified as a key determinant of the attenuated phenotype of Sabin 3 (a U-to-C reversion at nucleotide 2034 in the viral protein 3 (*VP3*) coding region, which caused an amino acid substitution in VP3–91: Phe to Ser) did not revert (Table 1). It is worth noting that although Henan VDPV recombined the sequence of type 1 in the *3D* region due to the occurrence of recombination, the attenuated site in the *3D* region (position 6203 in the 3D region, causing amino acid substitutions in 3D-73: Tyr to His) did not revert (Table 1).

**Table 1:** Two key neurovirulence determining sites revert in Henan aVDPV

Type	Region	Nucletide position	Sabin	CHN21006-1	CHN21006-2	CHN21006-3	Amino acid position	Sabin	CHN21006-1	CHN21006-2	CHN21006-3
Serotype 3	*5 ‘UTR*	472	U	C	C	C	**/**	**/**	**/**	**/**	**/**
	*VP3*	2034	U	U	U	U	430	Phe	Phe	Phe	Phe
	*VP1*	2493	C	U	U	U	583	Thr	Ile	Ile	Ile
Serotype 1	*3D*	6203	C	C	C	C	1824	His	His	His	His

*aVDPV* ambiguous vaccine-derived poliovirus, *UTR* untranslated region, *VP1* viral protein 1, *VP3* viral protein 3

### Antigenic divergence of Henan type 3 VDPVs

**Fig. 4:**
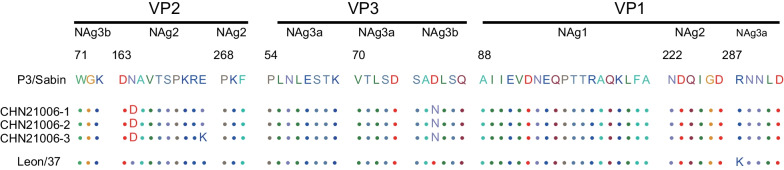
Alignment of amino acid residues of neutralizing antigenic (NAg) sites. NAg1 (VP1: 88–106), NAg2 (VP2: 163–172; VP2: 268–270; VP1: 222–227), NAg3a (VP3: 54–61; VP3: 70–74; VP1: 287–291), and NAg3b (VP2: 71–73; VP3: 75–80) were identified in Sabin 3 (GenBank accession no. AY184221), Henan type 3 VDPVs, and the type 3 wild poliovirus prototype strain P3/Leon/37 (GenBank accession no. K01392). *NAg* neutralizing antigenic, *VP1* viral protein 1, *VP2* viral protein 2, *VP3* viral protein 3

The Henan type 3 VDPVs had two shared amino acid replacement in the NAg sites, one is in NAg2 [viral protein 2 (VP2)–164: Asn to Asp, and], and the other is in NAg3b (VP3–77: Asp to Asn). Strain CHN21006-3 also had another amino acid replacement in NAg3b (VP2–172: Glu to Lys) (Fig. 4).

### Supplementary immunization activities (SIAs)

To prevent possible circulating VDPV, 54,916 children under 7 years of age were covered by SIAs performed throughout the whole county. It is worth mentioning that the bivalent OPV (bOPV, containing Sabin virus types 1 and 3) was used in SIAs, instead of the trivalent OPV (tOPV).

### Nucleotide sequence accession number

The full-length genome sequences of Henan type 3 VDPV strains CHN21006-1, CHN21006-2, and CHN21006-3 described in this study were deposited in the GenBank database under the accession numbers ON221307- ON221309.

## Discussion

This study reported a VDPV case detected by AFP case surveillance in Henan Province in 2017. After the case was identified as type 3 VDPV by the National Polio Laboratory, the emergency response of grade IV was immediately launched in Henan province. A timely emergency response was conducted, including an epidemiological investigation, and SIAs were performed in time to prevent the possible circulation of VDPV in the population and outside environment. Based on active searches of AFP cases, these preventive approaches proved to be effective and successful, and no poliovirus was detected in the close contacts and surrounding environment of the patient. In addition, through a comprehensive risk assessment, the risk of VDPV transmission was small, and cVDPV did not occur during the epidemic events.

In 2000, the WHO Western Pacific region, which includes China, declared that China had entered the stage of maintaining the polio-free status. In the process of eradicating polio and maintaining polio-free status, the occurrence of VDPV cases in some countries and regions in recent years is a newly emerging problem. For many years, massive OPV immunization campaigns worldwide have substantially reduced the number of cases of poliomyelitis caused by WPV. Nevertheless, challenges originating from low polio vaccine coverage could lead to the emergence of VDPVs or the reintroduction of WPV from disease-endemic countries, which might threaten the success of poliomyelitis eradication programs [2, 31]. Based on the characteristics of OPV, VDPV will inevitably appear as long as OPV are used. Meanwhile, poliomyelitis outbreaks caused by pathogenic VDPVs are primarily caused by low polio vaccine coverage. Low coverage enables interhuman circulation of polioviruses from the OPV strains and the genetic drift of the viruses, with the potential danger of subsequent reversion to neurovirulent phenotypes. If VDPV exists or causes a single case, it will not constitute a major threat to public health. However, once cVDPVs that cause disease outbreaks are present, they become a major public health emergency.

Reports of type 3 VDPV are relatively rare compared to those of types 1 and 2 VDPVs. In addition, only 11 records of type 3 VDPV sequences are available in GenBank as of April 2022. Both Chinese and global data show that type 2 VDPV account for the vast majority of all VDPVs. However, since the global switch from tOPV to bOPV in April 2016, the prevalence of types 1 and 3 VDPV may have changed [32].

A unique aspect of this case is that the patient had been vaccinated with four doses of IPV, and the serum neutralizing antibody in the serum for Sabin types 1 and 3 were 1:16 and 1:512, respectively. Since the titer of anti-type 3 poliovirus antibody was significantly higher than that of anti-type 1 poliovirus antibody, it is speculated that the patient was infected with type 3 VDPV from other asymptomatic infected individuals or from the environment, and the virus continued to replicate and be excreted for at least 41 days (from the date of the first stool sample collection to the date of the third stool sample collection). The presence of this kind of virus in the human population is a serious risk and undoubtedly poses a severe challenge in maintaining a polio-free status in China. At the same time, we reviewed the follow-up records of children who received the same batch of IPV as this child, and there were no records of adverse events following immunization with same batch the vaccine, indicating that this batch IPV itself was qualified.

How the virus infected this patient remains unclear. All stool samples collected from patients with AFP, meningitis, or hand, foot, and mouth disease in 2016–2017 were tested for virus isolation; however, no signs of poliovirus circulation were found before or after the isolation of CHN21006. Environmental surveillance was strengthened by collecting more wastewater samples from different areas of Zhumadian City; however, this did not lead to the isolation of similar viruses for the second time.

The emergence of the Henan VDPV has important implications for present and future polio immunization policies. This case report clearly showed that Henan type 3 VDPVs were aVDPVs, and the epidemiological data reported that they did not belong to cVDPVs or iVDPVs, that is, no other patients were found to have AFP associated with these VDPVs. Furthermore, no similar VDPVs could be isolated from their contacts. In addition, no signs of immunodeficiency, such as abnormal immunoglobulin levels or signs of abnormal T cell and B cell function, appeared in patients from whom VDPVs were isolated at the time of AFP presentation. Fortunately, no evidence of transmission of this VDPV among the general population was observed. Nevertheless, finding the source of the virus is difficult and remains unclear.

As the world approaches the goal of polio eradication, the WHO is developing corresponding strategies to minimize the risk of paralysis caused by poliovirus, including WPV and VDPVs. Stopping the use of type II OPV is an important part of the strategies in the later stages of polio eradication, thereby switching from tOPV to bOPV. Since May 1, 2016, China has begun to implement the sequential immunization program of one dose of IPV plus three doses of bivalent OPV (containing type 1 and 3 OPV). Since January 1, 2020, China changed its immunization strategy again and began the sequential immunization procedure of two doses of IPV plus two doses of bivalent OPV (containing type I and type III OPV). This program has a good protective effect, prevents the transmission of imported poliovirus, and reduces the incidence of VAPP and VDPV cases. As the last case of WPV 3 was observed in 2012, the WHO declares type 3 poliovirus eradicated after a 31-year campaign [33]. Under these circumstances, consideration should be given to hastening the global withdrawal of Sabin serotype 3 from OPV.

## Conclusions

Our study described the genetic characteristics of Sabin 3/Sabin 1 recombinant VDPV, which seems to have the characteristics of an iVDPV. Although the observed antigenic changes will help the virus escape vaccine-induced immunity in human populations, evidence of virus transmission was not observed. To the best of our knowledge, this is the first report of VDPV identified in the Henan province of China. The existence of this kind of virus in human population is a serious risk and poses a severe challenge in maintaining a polio-free status in China. Our results highlight the importance of maintaining a high-level vaccination rate and highly sensitive AFP case surveillance system in intercepting VDPV transmission.

## Supplementary Information

**Additional file 1: Table S1.** Nucleotide and amino acid substitutions in the Henan type 3 VDPVs compared to Sabin strains. *UTR* untranslated region, *VP1* viral protein 1, *VP3* viral protein 3.

## Data Availability

The full-length genome sequences of Henan type 3 VDPV strains CHN21006-1, CHN21006-2, and CHN21006-3 described in this study were deposited in the GenBank database under the accession numbers ON221307- ON221309. The datasets used and/or analysed during the current study are available from the corresponding author on reasonable request.

## Abbreviations

VDPV: Vaccine-derived poliovirus
AFP: Acute flaccid paralysis
WHO: World Health Organization
WPV: Wild-type poliovirus
OPV: Oral polio vaccine
IPV: Inactive polio vaccine
VAPP: Vaccine-associated paralytic poliomyelitis
VP1: Viral protein 1
VP2: Viral protein 2
VP3: Viral protein 3
UTR: Untranslated region
cVDPVs: Circulating vaccine-derived polioviruses
iVDPV: Immunodeficient vaccine-derived poliovirus
aVDPV: Ambiguous vaccine-derived poliovirus
ITD: Intratypic identification
RD cell: Human rhabdomyoma cell
L20B cell: Mouse cell line expressing the gene for human cellular receptor for poliovirus
rRT-PCR: Real-time polymerase chain reaction
SIAs: Supplemental immunization activities
CDC: Center for Disease Control and Prevention
MEGA: Molecular Evolutionary Genetics Analysis
NAg: Neutralizing antigenic
bOPV bivalent OPV: TOPV: trivalent OPV
