# Supplementary material for: Epidemiological survey and genetic characterization of type 3 vaccine-derived poliovirus isolated from a patient with four doses of inactivated polio vaccine in Henan Province, China
Source: Infect Dis Poverty. 2022 Dec 14;11:124. doi: 10.1186/s40249-022-01028-1 (PMC9749236; doi:10.1186/s40249-022-01028-1)
Supplement: Supplementary file 1 — Additional file 1: Table S1. Nucleotide and amino acid substitutions in the Henan type 3 VDPVs compared to Sabin strains. UTR untranslated region, VP1 viral protein 1, VP3 viral protein 3. [file 40249_2022_1028_MOESM1_ESM.docx]

|  | Region | Nucleotide |  |  |  |  | Amino acid |  |  |  |  |
| --- | --- | --- | --- | --- | --- | --- | --- | --- | --- | --- | --- |
|  |  | Position | Sabin | CHN21006-1 | CHN21006-2 | CHN21006-3 | Position | Sabin | CHN21006-1 | CHN21006-2 | CHN21006-3 |
| Sabin 3 | 5‘UTR | 28 | C | U | U | U |  |  |  |  |  |
|  |  | 60 | U | C | C | C |  |  |  |  |  |
|  |  | 69 | A | G | G | G |  |  |  |  |  |
|  |  | 472 | U | C | C | C |  |  |  |  |  |
|  |  | 667 | U | C | C | C |  |  |  |  |  |
|  |  | 703 | A | A | A | G |  |  |  |  |  |
|  | VP2 | 1071 | U | U | U | A | 109 | F | F | F | Y |
|  |  | 1412 | A | G | G | G | 223 | K | E | E | E |
|  |  | 1439 | A | G | G | G | 232 | N | D | D | D |
|  |  | 1463 | G | G | G | A | 240 | E | E | E | K |
|  |  | 1559 | A | U | U | U | 272 | S | C | C | C |
|  |  | 1592 | A | U | U | U | 283 | L | M | M | M |
|  |  | 1693 | U | C | C | C |  |  |  |  |  |
|  |  | 1726 | C | U | U | U |  |  |  |  |  |
|  | VP3 | 1780 | C | U | U | U |  |  |  |  |  |
|  |  | 1837 | C | C | C | U |  |  |  |  |  |
|  |  | 1991 | G | A | A | A | 416 | D | N | N | N |
|  |  | 2095 | C | C | C | U |  |  |  |  |  |
|  |  | 2153 | C | U | U | U |  |  |  |  |  |
|  |  | 2222 | G | G | A | A | 493 | V | V | U | U |
|  |  | 2223 | U | U | C | C |  |  |  |  |  |
|  |  | 2464 | U | C | C | C |  |  |  |  |  |
|  | VP1 | 2493 | C | U | U | U | 583 | T | I | I | I |
|  |  | 2506 | A | G | G | A |  |  |  |  |  |
|  |  | 2637 | C | U | U | U | 631 | A | V | V | V |
|  |  | 2651 | G | A | A | A | 636 | V | I | I | I |
|  |  | 2728 | C | U | U | U |  |  |  |  |  |
|  |  | 2767 | C | U | U | U |  |  |  |  |  |
|  |  | 2884 | A | G | G | G |  |  |  |  |  |
|  |  | 2972 | A | A | A | C | 744 | K | K | K | C |
|  |  | 2989 | C | C | C | U |  |  |  |  |  |
|  |  | 3106 | C | U | U | U |  |  |  |  |  |
|  |  | 3190 | A | G | G | G |  |  |  |  |  |
|  | 2A | 3727 | U | C | C | C |  |  |  |  |  |
|  | 2B | 3838 | U | U | U | C |  |  |  |  |  |
|  | 2C | 4195 | A | A | A | G |  |  |  |  |  |
|  |  | 4312 | U | C | C | C |  |  |  |  |  |
|  |  | 4363 | G | G | G | A |  |  |  |  |  |
|  |  | 4384 | C | U | U | C |  |  |  |  |  |
|  |  | 4663 | U | U | U | C |  |  |  |  |  |
| Sabin 1 |  | 4953 | G | A | A | A | 1403 | R | K | K | K |
|  |  | 4975 | U | U | U | C |  |  |  |  |  |
|  |  | 5032 | C | U | U | U |  |  |  |  |  |
|  |  | 5093 | U | C | C | C |  |  |  |  |  |
|  | 3A | 5119 | A | A | A | G |  |  |  |  |  |
|  |  | 5356 | A | G | G | G |  |  |  |  |  |
|  | 3C | 5461 | U | U | U | C |  |  |  |  |  |
|  |  | 5521 | C | C | C | U |  |  |  |  |  |
|  |  | 5530 | G | G | G | A |  |  |  |  |  |
|  |  | 5680 | A | A | A | G |  |  |  |  |  |
|  |  | 5974 | U | U | U | C |  |  |  |  |  |
|  | 3D | 6073 | U | C | C | C |  |  |  |  |  |
|  |  | 6265 | C | U | U | U |  |  |  |  |  |
|  |  | 6437 | C | U | U | U |  |  |  |  |  |
|  |  | 6725 | G | A | A | A | 1994 | E | K | K | K |
|  |  | 6772 | C | U | U | U |  |  |  |  |  |
|  |  | 6898 | A | G | G | G |  |  |  |  |  |
|  |  | 6905 | C | U | U | U |  |  |  |  |  |
|  |  | 6952 | C | C | C | U |  |  |  |  |  |
|  |  | 7060 | U | C | C | C |  |  |  |  |  |
|  |  | 7198 | U | U | U | C |  |  |  |  |  |
|  |  | 7351 | U | C | C | C |  |  |  |  |  |
